# IS LIFETIME ABUSE FORGIVABLE IN OLD AGE?

**DOI:** 10.1093/geroni/igad104.1857

**Published:** 2023-12-21

**Authors:** Tova Band-Winterstein, Ksenya Shulyaev, Zvi Eisikovits

**Affiliations:** University of Haifa, Haifa, Hefa, Israel; University of Haifa, Haifa, Hefa, Israel; University of Haifa, Haifa, Hefa, Israel

## Abstract

Old age is characterized by reflection and retrospective examination of the multiple meanings of various life experiences including lifetime abuse. Forgiveness was found to have salutary effects on older adults’ wellbeing. To enhance understanding concerning the place and role of forgiveness in the reflective process in later life we performed a secondary analysis of data from four previous studies using in in-depth, semi-structured interviews (N=78) with older women who survived abuse. Concepts developed deductively from reviewing the literature contributed to an inductive thematic analysis of the interviews. Three major themes emerged from this procedure: (1) The dimensions of forgiving: The victim as subject. Movement towards forgiveness occurs along three dimensions: painful memories with negative emotions, commitment to social obligations and life circumstances. (2) Being forgiven: Between lost forgiveness and hope. This theme related to the boundaries between the roles of being a victim and a victimizer simultaneously and describes the hope and willingness of survivors to be forgiven by closely related persons. (3) Self-forgiveness and the aging self. Forgiving self can be conceptualized from total renunciation due to exhaustion from lifetime abuse to full recognition of self-accepting forgiveness resulting from decision-making based on strength. While recognizing the salutary effects of forgiveness, we cannot disregard that this is not a universally desirable process. The dimension of forgiveness needs to be included in the study of abuse throughout the old person’s life-course to better understand its complexity Such view should substitute the either/or approach based on the dichotomy of “forgiveness” or “unforgiveness”.

